# The Emerging Role of Polo-Like Kinase 1 in Epithelial-Mesenchymal Transition and Tumor Metastasis

**DOI:** 10.3390/cancers9100131

**Published:** 2017-09-27

**Authors:** Zheng Fu, Donghua Wen

**Affiliations:** Department of Human and Molecular Genetics, VCU Institute of Molecular Medicine, VCU Massey Cancer Center, School of Medicine, Virginia Commonwealth University, School of Medicine, Richmond, VA 23298, USA; donghua.wen@vcuhealth.org

**Keywords:** PLK1, EMT, tumor invasion and metastasis, drug resistance, cancer therapy

## Abstract

Polo-like kinase 1 (PLK1) is a serine/threonine kinase that plays a key role in the regulation of the cell cycle. PLK1 is overexpressed in a variety of human tumors, and its expression level often correlates with increased cellular proliferation and poor prognosis in cancer patients. It has been suggested that PLK1 controls cancer development through multiple mechanisms that include canonical regulation of mitosis and cytokinesis, modulation of DNA replication, and cell survival. However, emerging evidence suggests novel and previously unanticipated roles for PLK1 during tumor development. In this review, we will summarize the recent advancements in our understanding of the oncogenic functions of PLK1, with a focus on its role in epithelial-mesenchymal transition and tumor invasion. We will further discuss the therapeutic potential of these functions.

## 1. Introduction

**Figure 1:**
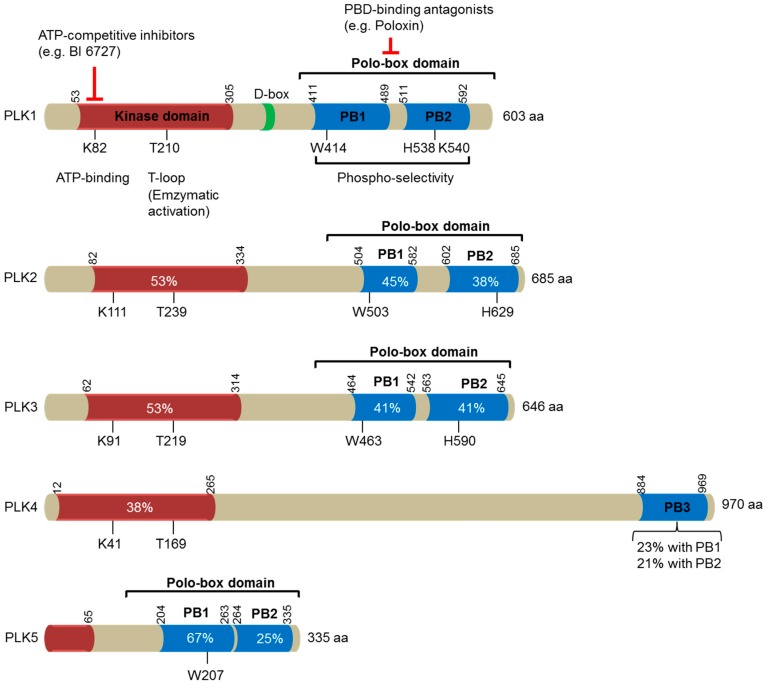
A schematic diagram illustrating the domain structures of the human polo-like kinase (PLK) family of proteins (PLK1-5). The number of amino acids in each family member is indicated on the right. The location of the kinase domains is shown in orange, whereas the polo-box domains (PBD), made of two polo-boxes (PB), are represented in blue. These two domains are separated by the interdomain linker, which comprises a destruction box (D-Box) indicated in green. The numbers indicate the first and the last residues of these domains in human PLKs. Residues that are essential for ATP-binding and enzymatic activation (T-loop) within the kinase domains, and for phosphoselectivity within the polo-box domains, are depicted. Sequence identities with the corresponding domains in PLK1 are provided in percentages. Two distinct strategies for targeting PLK1 are included: ATP-competitive inhibitors targeting the catalytic activity of PLK1, and PBD-binding antagonists competitively inhibiting the function of PBD.

Polo-like kinases (PLKs) belong to the polo subfamily of serine/threonine kinases, which are highly evolutionarily conserved, from yeast to humans [1]. PLKs have emerged as important regulators for cell cycle progression, cell proliferation, differentiation, and adaptive responses (see reviews; [1,2,3]). The prototypic founding member of the family was identified in *Drosophila melanogaster* in 1988 and was named “Polo” since the knockout of the gene induced abnormal spindle poles during mitosis [4]. Only one Plk has been reported in the genomes of *Drosophila* (Polo), budding yeast (Cdc5) and fission yeast (Plo1) [2], whereas vertebrates have many PLK family members [2]. In humans, five PLK members (PLK1-PLK5) have been identified and exhibit differential tissue distributions and distinct functions with no or partial overlap in substrates [1,2,5,6] (Figure 1). Among the human PLKs, PLK1 has been most extensively studied.

Sharing a similar domain topology with other PLKs, full-length PLK1 is composed of an N-terminal serine/threonine kinase domain and the characteristic polo-box domain (PBD) in the C-terminus [7] (Figure 1). The PBD is comprised of two polo boxes, polo box 1 and polo box 2, which fold together to form a functional PBD. The PBD binds phosphorylated serine/threonine motifs in PLK1’s substrates. The optimal binding motif of its substrates is Ser-[pSer/pThr]-[Pro/X], in which X represents any amino acid [8,9]. By binding with such motifs on its substrates, the PBD brings the enzyme to an array of substrates found at different subcellular structures, including centrosomes, kinetochores, the mitotic spindle, and the midbody. This confers diversity to PLK1’s function and allows exquisite regulation of the cell cycle [2,10]. A PBD mutant (H538A, K540M) that is deficient in phospho-binding delocalizes PLK1 and disrupts its function [11]. PLK1 also interacts with some of its binding partners in a phospho-independent or PBD-independent manner. For instance, aurora borealis (Bora), aurora kinase A activator, was reported to be capable of binding to a PLK1 deletion mutant that lacks the PBD [12]. In addition to the role of the PBD in interacting with PLK1’s substrates, the PBD also modulates PLK1’s kinase activity through intramolecular interaction [13,14]. The PBD inhibits the kinase domain by reducing its flexibility. Reciprocally, the kinase domain induces a conformational alteration of the PBD that renders it less capable of interacting with its binding targets. Phosphopeptide binding or activational phosphorylation of the T210 residue of PLK1 within the kinase activation loop relieves the inhibitory intramolecular interaction [9,15].

PLK1 mediates almost every stage of cell division, including mitotic entry, centrosome maturation, bipolar spindle formation, chromosome congression and segregation, mitotic exit, and cytokinesis execution [2]. In addition to its canonical role in mitosis and cytokinesis, recent studies suggest that PLK1 may have other important functions such as regulation of microtubule dynamics, DNA replication, chromosome dynamics, p53 activity, and recovery from DNA damage-induced G2 arrest [16,17].

PLK1 is overexpressed in a variety of human tumors, and its expression level often correlates with increased cellular proliferation and poor prognosis in cancer patients [18,19]. It has been suggested that PLK1 controls cancer development through multiple mechanisms that include the canonical regulation of mitosis and cytokinesis, as well as modulation of DNA replication and cell survival [20,21]. However, emerging evidence suggests that the oncogenic functions of PLK1 extend far beyond what is currently known [21]. Here, we will discuss the recent advances in the understanding of PLK1 as an oncogene, with a focus on its role in epithelial-mesenchymal transition (EMT) and tumor invasion. We will further discuss the potential for therapeutic targeting of these newly identified oncogenic actions of PLK1.

## 2. PLK1 in Tumor Development

### 2.1. PLK1 Expression in Human Cancers

**Figure 2:**
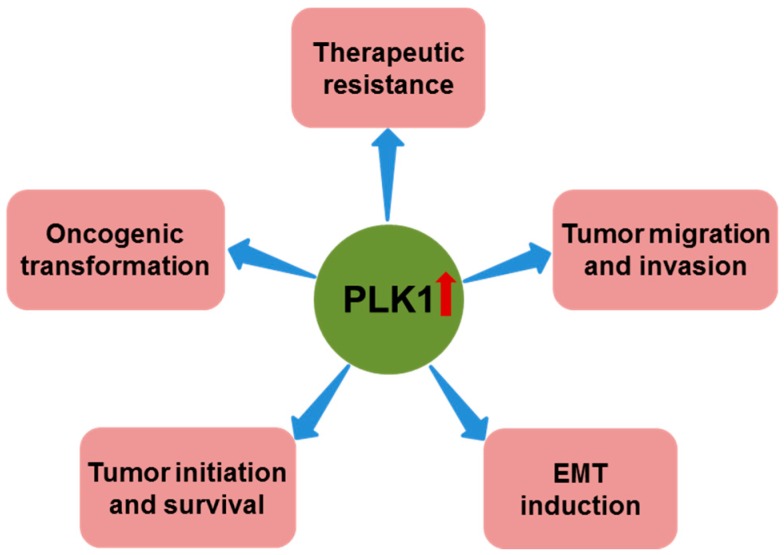
Role of polo-like kinase 1 (PLK1) overexpression in cancer. In addition to its role in promoting cancer cell proliferation and suppressing apoptosis, PLK1 overexpression has also been reported to have important roles in oncogenic transformation, tumor initiation and survival, epithelial-mesenchymal transition (EMT) induction, tumor migration and invasion, and therapeutic resistance.

Consistent with its role in mitosis, PLK1 is highly expressed in the late G2 and M phases of the cell cycle, and enhanced PLK1 activity is observed in cells with high mitotic rates, including tumor cells [22,23]. Increasing evidence suggests that PLK1 is closely linked to human cancer development. For example, *PLK1* is overexpressed in a variety of cancers, including prostate cancer [24], non-small cell lung cancer [25], head and neck cancer [26,27], esophageal and gastric cancer [28], melanoma [29], breast cancer [30], ovarian cancer [31], endometrial cancer [32], colorectal cancer [33], glioma [34], thyroid cancer [35], and hepatocellular cancer [36]. More importantly, its expression level often correlates with poor patient prognosis [19,24,26,27,28,29,37,38,39,40,41,42,43,44], suggesting that PLK1 is essential for tumorigenesis. Indeed, emerging evidence supports the notion that PLK1 is actively involved throughout the course of human cancer development [18,35,45,46,47,48,49] (Figure 2).

### 2.2. PLK1 and Oncogenic Pathways

A defining characteristic of cancer is the uncontrolled, abnormal growth of cells [50]. Since PLK1 plays a major role in regulating the cell cycle and maintaining genomic stability, it is believed that PLK1 controls cancer development through multiple mechanisms, including classic regulation of mitosis and cytokinesis, as well as response to cellular stress and cell survival [20,21].

However, recent studies show that PLK1 is capable of contributing to carcinogenesis through interconnections with multiple cancer-associated pathways. Several interacting partners of PLK1 have been identified that are encoded by tumor suppressor genes and oncogenes. For instance, the tumor suppressor *p53* is considered to be the “guardian of the genome” and plays an important role in antiproliferation. Recent studies suggest that PLK1 has the ability to control p53’s activity through multiple pathways: (1) PLK1 phosphorylates and inhibits *p53*-dependent transcriptional activation as well as p53’s pro-apoptotic activity [51]; (2) PLK1 phosphorylates MDM2, an E3 ubiquitin ligase for p53, to promote p53 turnover [52,53]; (3) PLK1-mediated phosphorylation of S718 on Topors, a ubiquitin and SUMO E3 ligase, inhibits Topors-mediated SUMOylation of p53 and enhances ubiquitin-mediated degradation of p53 [53]; (4) PLK1 also phosphorylates G2 and S-phase-expressed 1 (GTSE1), resulting in GTSE1’s translocation into the nucleus, where it binds to and shuttles p53 out of the nucleus for degradation [54].

Phosphatase and tensin homologue (PTEN) is one of the most commonly disrupted tumor suppressors in human cancers [55]. PLK1 has been identified as an important regulator of PTEN. PLK1 catalytic activity has been shown to phosphorylate PTEN near its C-terminal tail, which contributes to the mitotic function of PTEN [56]. Moreover, Li and colleagues showed that PLK1 phosphorylates PTEN and Nedd4-1, an E3 ubiquitin ligase of PTEN, which leads to the inactivation of PTEN and activation of the phosphatidylinositol 3-kinase (PI3K) pathway, thereby facilitating aerobic glycolysis and promoting tumorigenesis [57]. Analyses using prostate cancer cell lines, a prostate-specific PTEN-deletion mouse model, and a xenograft mouse model revealed that PLK1 is critical for PTEN-depleted cells to adapt to mitotic stress for survival, which assists the loss of PTEN-induced prostate cancer formation [58]. It has been reported that PLK1 also interacts with other tumor suppressors such as *CHK2* [59], *BRCA1/2* [60,61], *ATM* [62] and *ATR* [63], *BUB1B or BUBR1* [64,65], *CYLD* [66], *REST* [67], and *TSC1/2* [68,69]. The imbalance between these interactions and the resulting deregulation of oncogenic pathways could contribute to cancer development.

While negatively regulating tumor suppressors, PLK1 also intensively interplays with numerous oncogenes [70]. For instance, the Forkhead box protein M1 (FoxM1) transcription factor is a major mitotic transcription factor that is required for the proliferation of normal cells [71]. However, FoxM1 is frequently overexpressed in a wide spectrum of human cancers [72]. More importantly, overwhelming evidence reveals that FoxM1 is implicated in different phases of cancer development, and all major hallmarks of cancer delineated by Hanahan and Weinberg [50]. Our previous studies showed that PLK1 directly interacts with and phosphorylates FoxM1, leading to the activation of FoxM1’s transcriptional activity. Activated FoxM1 then transcribes multiple mitotic regulators, including *PLK1*, which generate a positive feedback loop to further increase PLK1 levels and FoxM1 activity [73].

The *MYC* family of oncogenes contains three members (c-Myc, L-Myc, and N-Myc), which have been implicated in the genesis of specific human cancers [74]. Several studies have shown that PLK1 induces c-Myc accumulation by direct phosphorylation [75,76]. PLK1 can also stabilize N-Myc via the PLK1-Fbw7-Myc signaling circuit [77]. PLK1 binds to and phosphorylates the specificity factor Fbw7 of SCF^Fbw7^ ubiquitin ligase, which promotes Fbw7 auto-polyubiquitination and proteasomal degradation and in turn prevents SCF^Fbw7^-mediated degradation of N-Myc. Stabilized N-Myc further activates the transcription of PLK1, resulting in a positive feed-forward regulatory loop that strengthens N-Myc-regulated oncogenic programs [77].

### 2.3. PLK1 and Oncogenic Transformation

The constitutive expression of *PLK1* in NIH/3T3 cells causes oncogenic foci formation and is tumorigenic in nude mice [78]. In contrast, depleting PLK1 in U2OS cells abrogates anchorage-independent growth [79]. These results highlight PLK1 as a possible driver of oncogenic transformation, although it remains unclear whether PLK1 itself is sufficient to induce tumor development. The oncogenic transformation potential of PLK1 has recently been documented in human cells. Our recent studies show that *PLK1* overexpression in human prostate epithelial cells leads to cellular transformation in vitro and promotes tumor formation in NOD/SCID/γ_c_^null^ (NSG) mice, which provides convincing evidence that PLK1 is directly involved in neoplastic transformation, and that PLK1 has a tumor-promoting role in the prostate [47]. 

### 2.4. PLK1 and EMT

A recent study from our group revealed an important additional function of PLK1 [47]. We documented an interesting observation that *PLK1* overexpression in prostate epithelial cells causes the cells to change shape from an orthogonal epithelial cell morphology to a spindle-shaped fibroblast-like morphology, reminiscent of cells having undergone EMT. EMT is an important mechanism of tumor progression and metastasis [80,81]. It involves a loss of epithelial cell characteristics (cell–cell junctions, apicobasal cell polarity, and cobblestone morphology) and an acquisition of mesenchymal characteristics (fibroblast-like cell morphology, increased cell-matrix adhesions, and motility). On the molecular level, EMT can be easily recognized by the reduced expression of epithelial markers such as E-cadherin and some cytokeratin isoforms, and the elevated expression of mesenchymal markers such as N-cadherin and vimentin. Significantly, the loss of cell–cell contacts and the reorganization of the intracellular cytoskeleton during EMT result in increased cell migration and invasion [82], which allows cells to invade the surrounding stroma and vasculature, thereby leading to tumor dissemination and metastases [83]. In addition, EMT enables cancer cells to avoid apoptosis, anoikis, and oncogene addiction [84].

Indeed, forced overexpression of PLK1 in prostate epithelial cells led to the downregulation of epithelial markers (E-cadherin and cytokeratin 19) and upregulation of mesenchymal markers (N-cadherin, vimentin, fibronectin, and SM22) [47]. The switch from epithelial to mesenchymal markers did not depend on a specific stage of the cell cycle. Importantly, *PLK1* overexpression in prostate epithelial cells disrupted the localization of E-cadherin, β-catenin, and junctional adhesion molecule (JAM)-A in areas of cell-cell contacts, which are indicative of the profound disassembly of adherens and tight junctions. In addition, this was accompanied by the dramatic reorganization of the actomyosin cytoskeleton manifested by the redistribution of non-muscle myosin IIB from perijunctional F-actin bundles into basal stress fibers. A comparison of EMT induction in cells expressing wild-type, constitutively active, or kinase-defective PLK1 suggests that a PLK1-mediated phosphorylation event contributes to the induction of EMT in prostate epithelial cells [47]. The role of PLK1 in EMT induction was further substantiated by the observation that PLK1 downregulation in metastatic prostate cancer cells enhances epithelial characteristics [47]. Moreover, an androgen-refractory cancer of the prostate (ARCaP) model was adopted for further validation [47]. ARCaP cells were derived from the ascites fluid of an 83-year-old Caucasian man diagnosed with metastatic prostate cancer [85]. Epithelium-like ARCaP_E_ cells and mesenchymal-like ARCaP_M_ cells are sublines of ARCaP cells that were isolated by single-cell dilution cloning [86]. Interestingly, PLK1 is not only differentially expressed and activated in these two cell lines (higher in the highly metastatic ARCaP_M_ cells and lower in the less metastatic ARCaP_E_ cells), it also controls the switch between EMT and mesenchymal-to-epithelial transition (MET) in those two cell lines (EMT induction in ARCaP_E_ cells upon PLK1 overexpression, and MET induction in ARCaP_M_ cells with PLK1 downregulation). Taken together, these results convincingly established a novel function of PLK1 as a critical regulator of EMT in prostate cancer.

**Figure 3:**
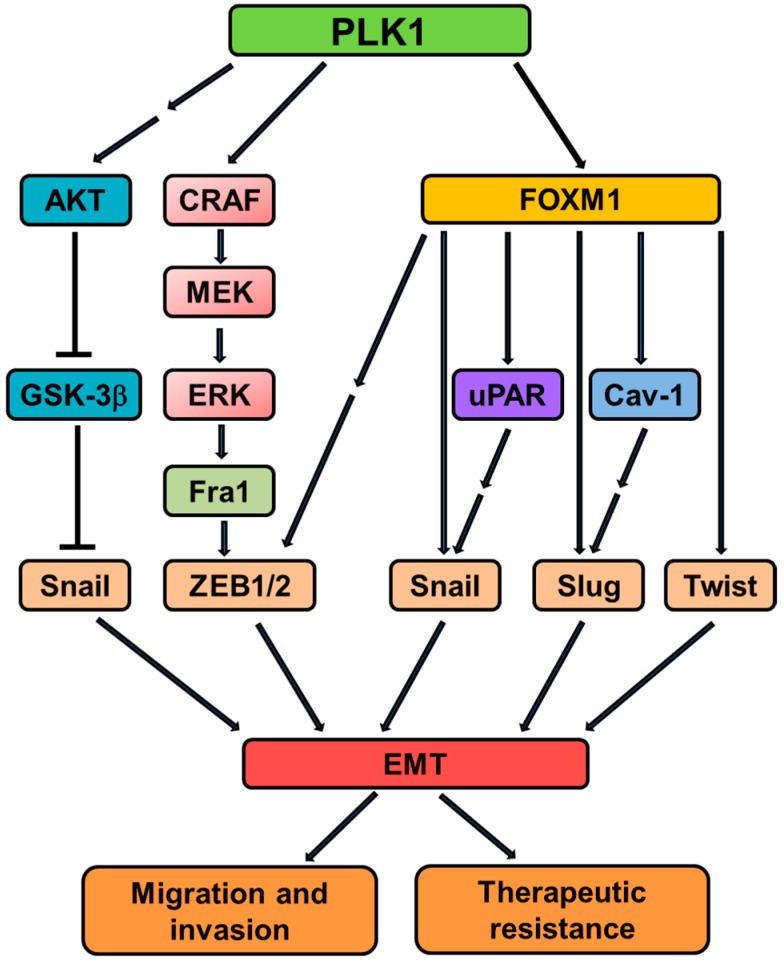
An overview of signaling cascades involved in PLK1-induced EMT. PLK1 activates the MAPK pathway by directly binding and phosphorylating CRAF. The activated MAPK pathway causes transcriptional upregulation of Fra1, which in turn triggers the accumulation of ZEB1/2, thus orchestrating the transcriptional network necessary for the EMT program. PLK1 also induces EMT through AKT or FoxM1-dependent pathways. Together, these signaling events contribute to EMT induction and associated events (such as invasion and therapeutic resistance) in tumor cells overexpressing PLK1.

Subsequently, the molecular mechanism underlying PLK1-mediated EMT was investigated [47]. We demonstrated that CRAF a member of the Raf kinase family of serine/threonine-specific protein kinases, is a physiological substrate of PLK1. CRAF consists of an N-terminal regulatory domain and a C-terminal catalytic domain. PLK1 directly interacts with and phosphorylates CRAF at S338 and S339 (the critical activating phosphorylation sites), resulting in CRAF activation. The activated CRAF undergoes autophosphorylation of S621, which hinders the proteasome-mediated degradation of CRAF, and thereby generates a positive feedback loop, leading to a further increase in the level and activity of CRAF. This activation event triggers the activation of downstream MEK1/2-ERK1/2 signaling in prostate epithelial cells overexpressing PLK1. Through a series of biochemical analyses, the events between PLK1-triggered MAPK signaling and EMT induction were elucidated [47]. ERK activation stimulates Fra1 expression; Fra1 belongs to the *Fos* gene family, whose protein products can dimerize with proteins of the JUN family, thereby forming the transcription factor complex AP-1. The ectopic expression of Fra1 in epithelioid cells resulted in morphologic changes that resembled fibroblastoid conversion, and increased motility and invasiveness [87]. Fra1 has been implicated as a potent regulator of anti-apoptosis, cell motility, and invasion in a variety of tumor cell types [88,89]. Enhanced expression of Fra1 then leads to the transcriptional activation of zinc finger E-box binding homeobox (ZEB) 1 and 2, two key transcription factors in EMT that orchestrate the EMT program [47]. Later, Cai et al. documented that PLK1 promotes EMT in gastric carcinoma cells through regulation of the AKT pathway [90], which suggests that regulating EMT is a general, and not a cell-type specific, function of PLK1, and the underlying mechanisms of PLK1-dependent EMT induction may vary dramatically from one setting to another. In addition to direct regulation, PLK1 may indirectly contribute to EMT induction through its substrates. For instance, FoxM1 has been linked to EMT in various tumor types, including pancreatic cancer [91,92], breast cancer [93], prostate cancer [94], gastric cancer [95], and lung cancer [96]. Several EMT regulators, such as Snail [97], Slug [93], and Twist [98] have been documented as direct targets of FoxM1. Given the aforementioned PLK1–FoxM1 regulatory circuit [73], it is likely that PLK1 may contribute to the EMT process by directly binding to and phosphorylating FoxM1, resulting in the activation of its transcriptional activity (Figure 3).

## 3. PLK1 in Tumor Invasion and Metastasis

Elevated PLK1 expression has been associated with an increased invasiveness of colorectal, breast, renal, and thyroid cancer cells [45,46,47,48,49]. PLK1 inhibition using either siRNA or pharmacological inhibitors caused significant reductions in the invasiveness of glioblastoma, bladder carcinoma, renal cell carcinoma, anaplastic thyroid carcinoma, and colorectal cancer cells [45,48,49,99,100]. Our recent study has provided direct evidence of the pro-invasive activity of PLK1 in tumor progression [47]. PLK1 was differentially expressed and/or activated in prostate cancer cells (higher in metastatic prostate cancer cell lines and lower in non-metastatic cell lines) [47]. In addition to EMT induction, *PLK1* overexpression in prostate epithelial cells led to enhanced motility and invasiveness, as manifested by wound-healing scratch and Transwell invasion analyses [47]. The results were further validated by monitoring the random movement of the cells using time-lapse video microscopy and cell tracking, which indicated that PLK1 directly regulates the velocity of epithelial cell migration, independently of its effects on other cellular processes. Interestingly, NOD/SCID/γ_c_^null^ (NSG) mice engrafted with PLK1-overexpressing prostate epithelial cells developed not only primary tumors, but also lung micrometastases, which suggests that *PLK1* overexpression not only leads to the oncogenic transformation of prostate epithelial cells, but may also drive prostate cancer metastasis [47]. Consistently, PLK1 downregulation in metastatic prostate cancer cells inhibited cell motility [47].

Both the profound disassembly of adherens and tight junctions and the dramatic reorganization of the actomyosin cytoskeleton were observed in prostate epithelial cells undergoing PLK1-mediated EMT [47]. Therefore, the following mechanisms by which EMT induction promotes prostate cancer cell motility were proposed: (1) disassembly of epithelial junctions that weaken intercellular adhesions, thereby allowing cell dissemination [101,102], and (2) rearrangement of the actomyosin cytoskeleton from epithelia-specific perijunctional bundles to basal stress fibers that are characteristic of mesenchymal cells. This rearrangement enhances cell–matrix adhesion and enables more efficient cell migration [103,104].

In line with these findings, Rizki et al. showed that PLK1 mediates invasion through vimentin and β1 integrin in breast cancer cells, which is independent of its mitotic function [46]. PLK1 phosphorylates vimentin on S82, which regulates cell surface levels of β1 integrin and thereby promotes the invasiveness of breast cancer cells [46]. In addition, it has been reported that the downregulation of PLK1 in thyroid cancer cells led to a significant decrease in CD44v6, matrix metalloproteinase (MMP)-2, and MMP-9, which are all key players in tumor invasion and metastasis [49].

## 4. PLK1 as a Key Target for Cancer Therapy

PLK1 has been reported as widely overexpressed in tumor samples from cancer patients, and its overexpression has been validated as a biomarker of poor prognosis in a variety of human cancers [18,19]. Importantly, several studies have shown that inhibiting PLK1 expression or function by antibodies, RNAi, or small molecule inhibitors leads to mitotic arrest and apoptotic cell death in a wide range of human cancer cells, and is sufficient to prompt tumor regression in mouse xenograft models [105]. In contrast, toxicity modeling of PLK1-targeted therapies using primary human cells and various organs of adult Plk1 RNAi mice reveals that normal cells can tolerate up to a ~80% reduction in PLK1 level [106]. Thus, it has been repeatedly proposed that PLK1 could be a particularly attractive target for anti-cancer drug discovery [107]. Over the years, PLK1 has been the subject of an extensive effort in developing anti-mitotic agents that primarily target fast-growing mitotic cancer cells while leaving normal cells unscathed. To date, a large number of anti-PLK1 agents have been developed and tested under various preclinical and clinical settings, and several of them are currently in clinical trials, with varying degrees of success (for a comprehensive review, see [108,109]).

There are two druggable domains of PLK1 that have been pursued extensively: the catalytic domain and the PBD domain [108,109] (Figure 1). Volasertib (BI6727, a dihydropteridine derivative; Boehringer Ingelheim) is the most advanced inhibitor in the class of ATP-competitive inhibitors directed against the catalytic activity of PLK1. Its anti-cancer efficacies have been evaluated and proven to be superior in multiple nude mouse xenograft models [110]. Considerably, volasertib has also shown significant clinical efficacies against advanced solid and hematologic cancers in phase I/II clinical trials. Subsequently, a phase III clinical trial in elderly patients with acute myeloid leukemia was undertaken. The initial outcome, however, turned out to be less than satisfactory (presented at the 21st Annual Congress of the European Hematology Association, 2016 [111]). One of the major problems associated with the currently available PLK1 ATP-competitive inhibitors is their low degree of selectivity against other kinases, and their toxicity could be partly due to their interference with other kinases [108]. In the case of volasertib, it inhibits PLK2 and PLK3, with similar IC50 values as PLK1 [110,112]. A new generation of anti-PLK1 agents that target the PBD domain of PLK are currently being tested pre-clinically and have demonstrated improved specificity towards PLK1 [113]. Among all of the published PBD-interfering compounds, the most potent and selective inhibitors of the PBD have been peptide-like molecules [114,115,116]. However, they are often associated with poor cell membrane permeability due to their large size and the presence of charged groups [115,117]. Therefore, further efforts towards anti-PLK1 drug discovery will need to find new compounds with increased potency and specificity and improved pharmacokinetic properties to achieve better clinical outcomes. 

The current rationale behind targeting PLK1 for anti-cancer therapy lies in its multifaceted functions throughout the cell cycle that target cancer’s sustaining proliferative signaling. Our recent study showed that *PLK1* overexpression induces EMT and promotes cell motility and invasiveness in human prostate epithelial cells; whereas the attenuation of *PLK1* expression reduces the invasiveness of human prostate cancer cells [47]. These novel findings not only provide mechanistic insight into the important role of *PLK1* overexpression in human cancer development and metastasis, but will also aid the advancement of the prevention and treatment of advanced prostate cancer human cancers. In this regard, PLK1 can serve as a molecular biomarker to improve the stratification of cancer patients at high-, intermediate-, or low-risk of metastatic progression. In addition, PLK1 inhibition could potentially be a promising strategy to prevent prostate cancer dissemination. Consistent with this notion, a recent study reported that PLK1 depletion, mediated by PLK1 siRNA delivered by an antioxidant nanoparticle platform, inhibits lung metastasis and prolongs overall survival in a mouse model of breast cancer metastasis [118]. PLK1 inhibition by a small molecule inhibitor hindered brain metastases and prolonged survival in a mouse model of breast cancer brain metastasis [119]. These encouraging findings provide a proof of concept to substantiate the hypothesis above. Since the pro-metastatic properties of *PLK1* overexpression were also reported in other different types of cancers, including breast and thyroid cancers [46,49], this suggests that targeting cancer’s ability to activate invasion and metastasis by PLK1 inhibition could be generalized to a growing list of a variety of cancers that have *PLK1* overexpression implicated in their metastatic progression. 

In recent years, EMT has also emerged to be a major driver of resistance to anti-cancer therapies, manifested not only in experimental models, but also in clinical settings [120]. Remnant cancer cells that survive after different types of therapies (including chemotherapy, molecularly targeted therapy, and immunotherapy), recurrently display signs of EMT activation [120]. Gene expression profiling of tumor samples revealed a strong correlation between an EMT gene signature and resistance to chemotherapy [121,122]. However, the mechanisms by which the activation of the EMT program triggers the development of resistance to therapeutics in cancer cells remain elusive. Nonetheless, these findings suggest that targeting cancer cells that have activated the EMT program might significantly improve the efficiency of therapeutic modalities in generating durable clinical responses. In this regard, targeting signaling pathways that are critical for the activation and subsequent maintenance of the EMT program have been demonstrated to be an effective therapeutic approach to prevent and/or reverse the EMT process, thereby overcoming therapeutic resistance [120]. As a major driving force of the EMT process in prostate cancer [47], PLK1 overexpression might contribute to therapeutic resistance to anti-cancer therapies through EMT. Indeed, PLK1 has been reported to be closely associated with drug resistance in cancer cells to a number of chemotherapy drugs, including doxorubicin, paclitaxel, and gemcitabine [108]. Therefore, the inhibition of PLK1 might reverse the drug resistance and increase sensitivity to chemotherapy. In this line of thinking, PLK1 inhibition has been reported to be effective in treating EGFR-inhibitor resistant non-small cell lung cancer through EMT [123,124]. Furthermore, PLK1 inhibition has been documented to enhance anti-cancer drug efficacy in a variety of types of human cancers [125,126,127,128]. Taken together, the emerging evidence for the novel oncogenic roles of PLK1 (ranging from neoplastic transformation, EMT induction, tumor invasion and metastasis, and therapeutic resistance) further highlights PLK1 as a fascinating anti-cancer target, and may substantially aid in developing and deploying anti-PLK1 therapeutics. It is reasonable to speculate that targeting PLK1 has the potential for multi-dimensional actions against cancer, and ultimately paves the way for curative cancer treatments.

## 5. Conclusions and Outlook

PLK1 is a fascinating multifaceted protein that targets many binding partners to ensure proper cell cycle progression and cell proliferation, and its deregulation contributes to the genesis of a broad range of human cancers. The differential requirement of PLK1 levels in cancer versus normal cells for survival makes PLK1 a particularly attractive target for anti-cancer drug discovery. Recently, a wealth of data has shed new light on the additional biochemical functions of PLK1 proteins and on the mechanisms through which they function in neoplastic transformation, tumor progression and dissemination, and the development of therapeutic resistance. The identification of the diverse roles of PLK1 throughout the course of tumor development highlights PLK1 as one of the most appealing anti-cancer drug targets.

Anti-PLK1 drug discovery has reached an advanced stage of development. A number of PLK1 inhibitors have been developed. The major problems commonly associated with currently available PLK1 inhibitors are insufficient specificity and cancer cell-selective killing. Further studies are needed to identify new compounds with increased potency and specificity and improved pharmacokinetic properties. Furthermore, there is no doubt that additional functions of PLK1 will be uncovered in the near future. Additional studies aimed at disclosing all of the molecular mechanisms of PLK1 signaling in cancers are needed to achieve the full therapeutic potential of an anti-PLK1 drug. In other words, better understanding of the oncogenic action of PLK1 overexpression will greatly facilitate the optimization of treatment regimens targeting PLK1 signaling to significantly enhance therapeutic efficacy.
